# Source Apportionment of Ambient Black Carbon during the COVID-19 Lockdown

**DOI:** 10.3390/ijerph17239021

**Published:** 2020-12-03

**Authors:** Ismail Anil, Omar Alagha

**Affiliations:** Environmental Engineering Department, College of Engineering, Imam Abdulrahman Bin Faisal University, East Campus, P.O. Box 1982, Dammam 34212, Saudi Arabia

**Keywords:** black carbon, COVID-19, SARS-CoV-2, lockdown, source apportionment

## Abstract

Black carbon (BC) particles being emitted from mobile and stationary emission sources as a result of combustion activities have significant impacts on human health and climate change. A lot of social activities have been halted during the COVID-19 lockdowns, which has evidently enhanced the ambient and indoor air quality. This paper investigates the possible emission sources and evaluates the meteorological conditions that may affect the dispersion and transport of BC locally and regionally. Ground-level equivalent BC (eBC) measurements were performed between January 2020 and July 2020 at a university campus located in Dammam city of the Kingdom of Saudi Arabia (KSA). The fossil fuel (eBC_ff_) and biomass burning (eBC_bb_) fractions of total eBC (eBC_t_) concentrations were estimated as 84% and 16%, respectively, during the entire study period. The mean eBC_bb_, eBC_ff_, and eBC_t_ concentrations during the lockdown reduced by 14%, 24%, and 23%, respectively. The results of statistical analyses indicated that local fossil fuel burning emissions and atmospheric conditions apparently affected the observed eBC levels. Long-range potential source locations, including Iraq, Kuwait, Iran, distributed zones in the Arabian Gulf, and United Arab Emirates and regional source areas, such as the Arabian Gulf coastline of the KSA, Bahrain, and Qatar, were associated with moderate to high concentrations observed at the receptor site as a result of cluster analysis and concentration-weighted trajectory analysis methods.

## 1. Introduction

The most valuable resource and future asset of any country is its people. Exposing them to low air quality is a considerable risk for their health and welfare [1,2,3,4]. Nowadays, an enormous consumption of non-renewable energy resources by industrial expansion, urbanization, and energy demand has been leading to the deterioration of air quality locally and globally. Black carbon (BC) particles are health-threatening pollutants to human health, particularly in urban cities and industrial areas [1,2,3,5,6,7,8]. It is known that BC particles have both anthropogenic and natural sources, and they are mainly linked to the incomplete combustion of fossil fuels and biomass burning [9,10,11].

Fine and coarse particulate matter fractions can suspend in the atmosphere for a long time and be transported over long distances [12,13]. BC particles act as a host to many chemical and physical species during their lifetime (4–12 days) in the atmosphere [14]. Thus, the SARS-CoV-2 virus, having a diameter of around 0.1 µm, might be carried by BC particles to remote areas [15]. Cluster analysis (CA) of backward air mass trajectories, potential source contribution function (PSCF), and concentration weighted trajectory (CWT) analyses have been widely practiced to investigate the potential source regions of atmospheric pollutants [3,12,16,17,18,19]. The PSCF method’s constraint is that the grid cells might indicate identical PSCF values when the concentrations of the targeted air pollutant are slightly or considerably above the selected criteria. Larger sources cannot be differentiated from moderate sources in the PSCF method. The CWT analysis could overcome the limitation of the PSCF in differentiating the degree of effects caused by source regions on receptor areas [20,21].

Several researchers have studied the monitoring, characterization, source apportionment, and health risk assessment of ambient BC in Africa [22,23,24,25], Antarctica [26,27,28,29,30], Asia [1,6,31,32,33,34], Australia [35,36], Europe [21,37,38,39,40,41], North America [42,43,44,45,46], and South America [47,48,49] throughout the past four decades. To date, a few studies have been conducted on the ambient BC particles in the Kingdom of Saudi Arabia (KSA). Lihavainen et al. [50] investigated the aerosol’s physical properties between November 2012 and February 2015 in Haddat Ash Sham village in the Makkah Province. The mean BC concentration in the study area was found to be 2.1 ± 2.5 µg/m^3^, and the BC emissions were linked to local sources. In another study, Nayebare et al. [51] monitored BC levels from 26 February 2014 to 27 January 2015 in the holy city of Makkah, and the mean BC concentration was reported as 2.2 ± 0.9 µg/m^3^. Their study revealed that the high BC concentrations were associated with local traffic emissions and long-range transport contributions. Bian et al. [52] researched the sources of carbonaceous aerosols from April to September 2012 in Riyadh, the capital of the KSA. The mean BC concentration was 2.1 ± 2.5 µg/m^3^ during their study period. The high BC concentrations were attributed mainly to emissions from oil fields, local traffic, cement production, and partially to long-range source locations, including the Arabian Gulf and regions around Iraq.

After the outbreak of the novel coronavirus (SARS-CoV-2) in Wuhan, China, on 31 December 2019, it has quickly spread outside of China through human-to-human transmission, and the World Health Organization classified this new disease of “COVID-19” as a global pandemic on 11 March 2020. At the time of this writing, 29 November 2020, the death toll of COVID-19 has crossed 1.4 million, and more than 62 million people have been infected, according to the WHO’s reports [53]. Besides its devastating global effects on public health, economy, daily habits, and social life, people around the world have witnessed remarkable improvements and recoveries in almost all compartments of the environment in virtue of imposed lockdown measures to stop the spread of the COVID-19 pandemic [54,55,56,57]. One of the most significant benefits of COVID-19 lockdowns was probably on the public’s enhanced quality-adjusted life years in parallel with the increased air quality reported for many regions of the world [58,59,60,61,62,63,64,65]. A few studies have been conducted to reveal the impacts of COVID-19 lockdown measures on ambient BC concentrations. Reported studies from the USA [66], Italy [67], China [68,69], and India [70] revealed that BC concentrations reduced within the range between 22% and 71% in response to lockdown measures to combat the COVID-19 pandemic.

The main goal of this work is to examine ambient BC levels and evaluate the meteorological conditions that may affect the atmospheric dispersion and transport of BC locally and regionally by using an hourly aethalometer dataset collected between January 2020 and July 2020 at a university campus located within the core of Dammam city. In addition to that, the study aims to investigate source apportionment of ambient BC in the Eastern Province of the KSA. Besides this, the local and long-range potential sources of the measured BC levels have been studied by using statistical methods and source apportionment models before, during, and after COVID-19 lockdown periods. The research hypothesis is to examine whether the COVID-19 lockdown measures influenced the observed BC concentrations or not. This research study is the first attempt to measure and evaluate the ground-level ambient BC concentrations in the region, and the results will remain as a background level for future BC studies.

## 2. Materials and Methods

### 2.1. Study Area and Monitoring Period

**Figure 1:**
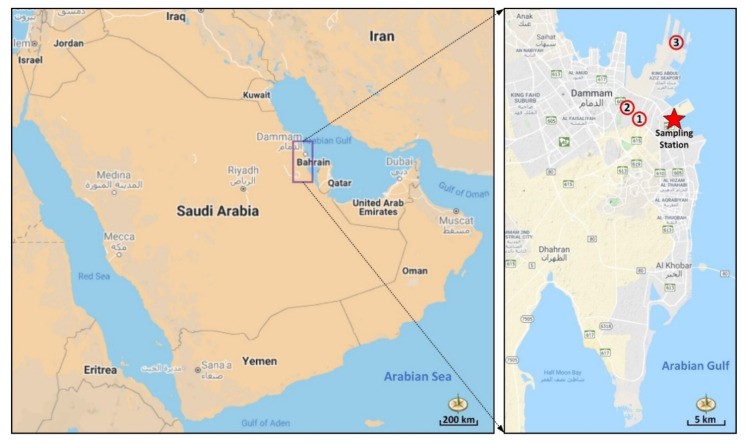
Location of the monitoring station and potential emission locations: (1) intersection of King Abdulaziz and King Fahd roads, (2) Industrial Area #1, and (3) King Abdulaziz Sea Port.

The Dammam metropolitan area, comprising of Dammam, Khobar, and Dhahran municipalities, is the sixth most populated city in the KSA, with a population of 1.1 million. Dammam has a hot desert climate, mild to warm in winter and hot in summer. The long-term mean annual temperature in Dammam is 26.8 °C, ranging between 20.1 and 34.5 °C. Northerly wind systems are dominant over the area, with a mean wind speed of 4 m/s. The precipitation regime of the Dammam metropolitan area has been classified as “arid”, since the annual total rainfall is 90 mm. The Air Quality Monitoring Station (AQMS) used in this study is located at the top of the engineering college building on the south campus of Imam Abdulrahman Bin Faisal University (26.3955 N, 50.1978 E) (Figure 1). An aethalometer for measuring BC mass concentrations is installed in the AQMS, which is 15 m above the sea level and 3 km away from the Arabian Gulf. Local anthropogenic emission spots, such as King Abdulaziz Sea Port (the second busiest and largest seaport of the KSA), industrial area #1, and two main arteries with high traffic density (King Abdulaziz and King Fahd roads), are present within the five-km perimeter of the AQMS. Jubail city, known as the largest industrial city in the world, and Qatif city, hosting the oil production field of Saudi Aramco (the Saudi national oil company) and Saudi Basic Industries Corporation (SABIC) petrochemical and chemical company, are the most significant regional industrial emission sources located in the northwestern part of the study area. Furthermore, Al Hofuf city in the southwestern section of the monitoring station accommodates the largest oil fields in the KSA and can be considered as a regional emission source. In this study, the measurements of BC mass concentrations and meteorological parameters were performed between 8 January and 2 July 2020. The obtained dataset was categorized into three phases: before lockdown (Pre-LD) (8 January–22 March), during lockdown (LD) (23 March–20 June), and after lockdown (Post-LD) (21 June–2 July) to find out any potential effects of COVID-19 lockdown countermeasures on ambient BC emissions.

### 2.2. Local Meteorology Data

**Figure 2:**
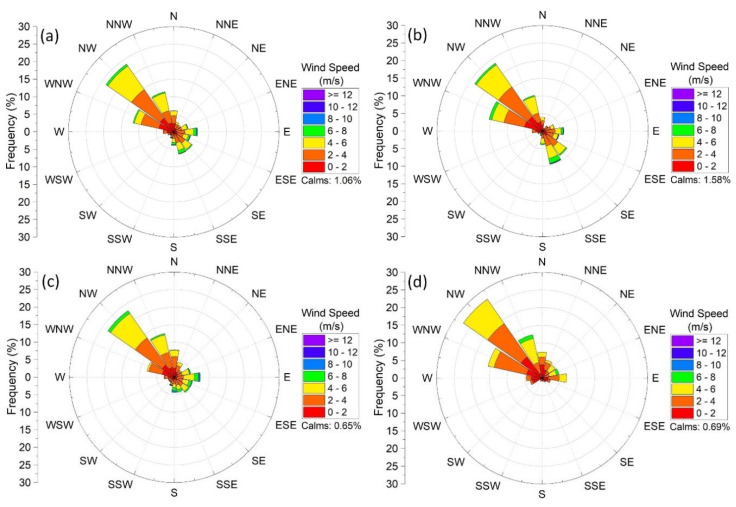
Wind-rose plots of the study area during (**a**) the entire study, (**b**) pre-lockdown (Pre-LD), (**c**) lockdown (LD), and (**d**) post-lockdown (Post-LD) periods.

The meteorological parameters, including temperature (°C), humidity (%), pressure (mbar), wind speed (m/s), rainfall (mm), wind direction (0–360°), and solar radiation (W/m^2^), used during this study were collected via a wireless meteorology station (Davis^®^ Vantage Pro2+, Hayward, CA, USA). The meteorology station is located next to the AQMS. The meteorology station is mounted 4 m above the roof to ensure that no obstacle impairs the measurements. The wind data statistical analysis revealed that the dominant wind directions during the study period originated from northwesterly sectors (Figure 2). The frequencies of the winds blowing from the WNW, NW, and NNW directions through the whole study, Pre-LD, LD, and Post-LD periods were found to be 47.1%, 49.5%, 43.9%, and 55.6%, respectively. The mean wind speeds for the NW prevailing direction during the Pre-LD, LD, and Post-LD periods were 3.3, 3.7, and 3.3 m/s, respectively.

### 2.3. Black Carbon Measurements and Data Treatment

The equivalent BC (eBC) mass concentrations were derived using a rack mount aethalometer (Magee Scientific^®^ Model AE-31), which is an optical absorption method [71,72]. The device installed in the AQMS has an ‘extended range’ inlet capable of measuring seven different wavelengths (370, 470, 520, 590, 660, 880, and 950 nm), as it is recommended for urban areas of moderate to high aerosol concentrations. A weatherproof PM_10_ inlet (M4121, Magee Scientific^®^, Berkeley, CA, USA) was installed on the top of the inlet tube. The PM_10_ inlet tube line with a length of two meters was covered with a temperature-controlled jacket heater to prevent water condensation. The possible loading effect due to the aethalometer’s filter-based optical method needs to be compensated to obtain accurate ambient eBC concentrations [73,74]. The procedure developed by Virkkula et al. [73] was employed to correct the reported raw eBC concentrations by the instrument using Equation (1):

eBC = eBC_raw_ (1 + k × ATN) (1)

where k is the compensation parameter and ATN is the optical attenuation value recorded by the instrument before and after each eBC reading.

The k-parameter may indicate seasonal variations depending on the aerosol properties [73,74]. In this study, the k-parameter for the nth filter spot was calculated by use of Equation (2), since the ATN values of the first measurement data of the new filter spots were all higher than zero. During this study, the mean k values were found to be 0.003 ± 0.003 at 370 nm and 0.005 ± 0.004 at 880 nm.

(2) $$k_{n}=\frac{eBC_{raw}\left(t_{n+1,first}\right)-eBC_{raw}\left(t_{n,last}\right)}{ATN\left(t_{n,last}\right)\times eBC_{raw}\left(t_{n,last}\right)-ATN\left(t_{n+1,first}\right)\times eBC_{raw}\left(t_{n+1,first}\right)}$$

where *t_n+_*_1,*first*_ is the time of the first measurement data for the next filter spot and *t_n,last_* is the time of the last measurement data for filter spot *n*.

The derivation of fossil fuel BC (BC_ff_) or biomass burning BC (BC_bb_) fractions is only an indicative datum when it is not supported by additional chemical composition measurements. BC_ff_ and BC_bb_ concentrations cannot precisely be calculated from the aethalometer measurements alone [71]. For this reason, BC_ff_ and BC_bb_ concentrations reported in this study indicate estimated concentrations. The estimation of biomass burning and fossil fuel fractions of the ambient eBC is dependent on the absorption coefficients (b_abs_) at the wavelengths of 370 and 880 nm, respectively. Initially, the attenuation coefficient of the analyzed particles (b_ATN_) should be calculated. In this study, the calculation of b_ATN_ and b_abs_ values was performed according to the methods developed by Hansen [75]. Equations (3) and (4) were used for the calculation of b_ATN_ and b_abs_, respectively:

(3) $$b_{\mathrm{ATN}}\equiv \frac{A}{Q}\;\frac{∆\mathrm{ATN}}{∆t}$$

where A is the aerosol collecting spot area of filter (1.67 cm^2^); Q is the sampling flow rate (5 L/min); ΔATN is the difference between the ATN values recorded by the instrument before and after each eBC reading, and Δt is the sampling time (5 min).

(4) $$b_{\mathrm{abs}}=\frac{b_{\mathrm{ATN}}}{{C\;R}_{\mathrm{ATN}}}$$

The multiple scattering parameter (C = 2.14) and a linear function of ln(ATN) (R_ATN_) in Equation (4) are defined as calibration factors, which are applied to b_ATN_ values to calculate b_abs_ values [37]. The shadowing effect of aerosols due to the filter overload is corrected by the R_ATN_ parameter, which is computed using Equation (5), where f is the free parameter and is found to be 1.14 and 1.08 for the wavelengths of 370 and 880 nm, respectively.

(5) $$R_{ATN}=\left(\frac{1}{f}-1\right)\frac{\ln\left(ATN\right)-\ln\left(10\%\right)}{\ln\left(50\%\right)-\ln\left(10\%\right)}+1$$

The concentrations of equivalent biomass burning fraction (eBC_bb_) and equivalent fossil fuel fraction (eBC_ff_) were predicted according to the model developed by Hansen [75], using the Beer–Lambert Law. The model assumes that b_abs_ is the sum of optical absorption coefficients of biomass burning (b_abs,bb_) and fossil fuel (b_abs,ff_) fractions, as given in Equations (6) and (7).

b_abs_(370 nm) = b_abs_(370 nm)_ff_ + b_abs_(370 nm)_bb_ (6)

b_abs_(880 nm) = b_abs_(880 nm)_ff_ + b_abs_(880 nm)_bb_ (7)

The b_abs,ff_ and b_abs,bb_ are computed for the wavelengths of 370 and 880 nm using Equations (8) and (9), respectively, by assuming that b_abs,bb_ is negligible at 880 nm [68,76]. The absorption exponents for fossil fuel (α_ff_) and for biomass (α_bb_) were used as 1 and 2, respectively.

(8) $$\frac{b_{\mathrm{abs}\left(370\;\mathrm{nm}\right)\mathrm{ff}}}{b_{\mathrm{abs}\left(880\;\mathrm{nm}\right)\mathrm{ff}}}={\left(\frac{370}{880}\right)}^{-\alpha_{\mathrm{ff}}}$$

(9) $$\frac{b_{\mathrm{abs}\left(370\;\mathrm{nm}\right)\mathrm{bb}}}{b_{\mathrm{abs}\left(880\;\mathrm{nm}\right)\mathrm{bb}}}={\left(\frac{370}{880}\right)}^{-\alpha_{\mathrm{bb}}}$$

The eBC_ff_ and eBC_bb_ concentrations can be calculated by using Equations (11) and (12), respectively, after obtaining the fossil fuel fraction (FF_fraction_) via Equation (10).

(10) $${\mathrm{FF}}_{\mathrm{fraction}}=\frac{b_{\mathrm{abs}\left(880\;\mathrm{nm}\right)\mathrm{ff}}}{b_{\mathrm{abs}\left(880\;\mathrm{nm}\right)}}$$

eBC_ff_ = FF_fraction_ × eBC (11)

eBC_bb_ = eBC − eBC_ff_ (12)

### 2.4. Statistical Analyses

Multivariate linear correlation analysis is a useful method to statistically determine the relationships between pollutants or other atmospheric factors influencing the air quality and to reveal the most significant parameters on the concentrations of atmospheric pollutants [12,43,77]. In this work, IBM^®^ SPSS^®^ statistics software (Ver. 24) was used to perform descriptive statistics, sample comparison, and multivariate linear correlation analyses for the hourly mean values of the measured eBC concentrations and meteorological parameters. Bivariate polar plots of concentrations have recently been used to reveal the collective impact of wind velocity and wind direction on air pollutants’ observed concentrations [78,79,80]. In this research, bivariate polar plots were drawn to illustrate the impact of wind components on hourly mean values of eBC concentrations through Pre-LD, LD, and Post-LD phases.

### 2.5. Source Apportionment Modeling

Cluster analysis is a statistical method to classify trajectories that are close to each other into particular groups. In a cluster analysis, similar trajectories are merged until the total variance is minimized and distinct groups are formed. The TrajStat air mass trajectory plugin (Ver. 1.4.9) of the MeteoInfo GIS (Geographic Information System) meteorology-dispersion model analysis software (Ver. 2.2.7) was employed to calculate the backward air mass trajectories. Furthermore, the previous model was used to perform cluster analysis, cluster statistics, and concentration weighted trajectory analysis [81]. The three-day backward trajectories arriving at 500-m height above ground level (AGL) to the receptor study site were computed for every 6 h throughout the whole studied period using the global data assimilation system (GDAS) meteorological data with half-degree resolution and 10,000 m (AGL) of model top height. Following these, the daily mean eBC concentrations were added to the respective trajectories.

In this study, the targeted percentage change criterion of 20% of the total spatial variance was selected to determine the optimum number of clusters [82]. Besides, the hourly time-averaged surface mass BC concentrations (within the domain of 10–45° N and 10–60° E and with a spatial resolution of 0.5° × 0.625°) were obtained from the Goddard Earth Observing System Model (GEOS-5) satellite observation database in netCDF data format, which was further processed by using the GIS interface of the MeteoInfo software. The satellite observation datasets were used to identify possible contributions from BC emission points to the clustered trajectories [2,83,84].

The CWT method has been widely practiced in investigating the relative importance of potential source areas. In a CWT analysis, each grid cell has a weighted average concentration for each pollutant related to the backward trajectories traveling over that grid cell, as given in Equation (13) [20,21]. Like the PSCF analysis, a point filter was employed as the last stage of CWT analysis to eliminate grid cells with limited endpoints [20]. As a result of the CWT analysis, weighted concentration zones indicate concentration gradients throughout potential source locations.

(13) $$C_{\mathrm{ij}}=\frac{1}{\sum_{l=1}^{n}t_{\mathrm{ijl}}}\overset{n}{\underset{l=1}{\sum }}c_{l}t_{\mathrm{ijl}}$$

where C_ij_ is the average weighted concentration in the grid cell (i,j); l is the trajectory index; n is the total number of trajectories; C_l_ is the measured concentration on the arrival of trajectory l; and t_ijl_ is the time spent by trajectory l in the grid cell (i,j).

## 3. Results and Discussions

### 3.1. Hourly Variations of BC Concentrations

Types of emission sources, emission rates, meteorological conditions, and atmospheric stability are the main factors that significantly impact the hourly variations of ambient BC concentrations [33,47,72,85,86]. Descriptive statistical parameters, such as mean, median, mode, standard deviation (S.D.), minimum (Min.), maximum (Max.), and percentiles, were calculated for both hourly mean values of meteorological parameters and eBC concentrations (Table 1). The relative differences between the mean and median values of temperature, humidity, wind speed, and pressure parameters during the Pre-LD, LD, and Post-LD periods were found to be less than 6%. The mean values of temperature, humidity, pressure, and solar radiation parameters along the LD changed by 59%, −24%, −1.1%, and 31%, respectively, as compared to the Pre-LD period. The changes in mean wind speed values during the LD and Post-LD periods in comparison with the Pre-LD stage were 3.1% and −12.5%, respectively. The comparison of wind speed and wind direction (Figure 2) suggests that the wind data were quite similar throughout Pre-LD and LD periods, while lower wind speeds during the Post-LD might result in higher eBC concentrations due to the atmospheric stability. During the whole study period, eBC_bb_, eBC_ff_, and eBC_t_ concentrations varied between 0.01 and 3.6, 0.12 and 13.4, and 0.25 and 14.2 µg/m^3^, respectively. The eBC concentrations measured within LD phase exhibited the lowest percentile (25th, 50th, and 75th) values. The independent sample comparison t-test was applied to hourly means, medians, and standard deviations of the eBC datasets of Pre-LD, LD, and Post-LD periods. The comparison test results revealed that the measured eBC concentrations (eBC_bb_, eBC_ff_, and eBC_t_) in each period were statistically different at the 95.0% confidence level.

**Figure 3:**
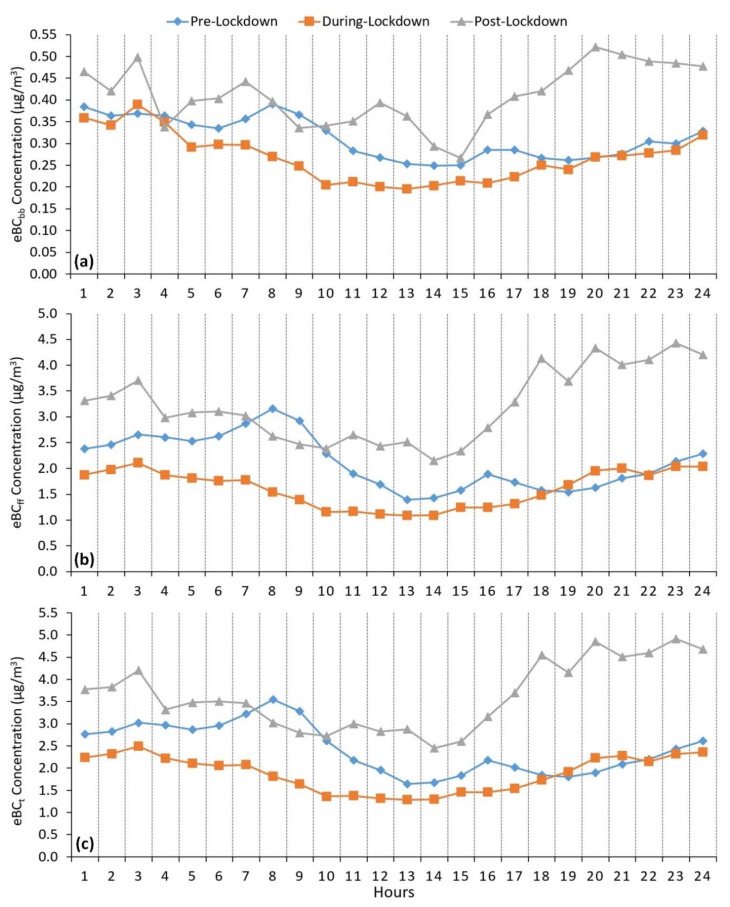
Hourly variations of equivalent black carbon (eBC) concentrations along Pre-LD, LD, and Post-LD phases: (**a**) eBC_bb_, (**b**) eBC_ff_, and (**c**) eBC_t_.

Hourly changes in eBC concentrations throughout the Pre-LD, LD, and Post-LD stages are depicted in Figure 3. Hourly variations in eBC_ff_ concentrations during Pre-LD, LD, and Post-LD indicated distinct bimodal distributions. Within the Pre-LD period, a significant and sharp peak between 5:00 am and 10:00 am was observed for the mean eBC_ff_ concentrations, which reached the maximum value of 3.2 ± 2.2 µg/m^3^ at 8:00 am and then gradually decreased to 1.4 ± 0.9 µg/m^3^ by 2:00 pm. Thereafter, eBC_ff_ concentrations demonstrated a minor peak between 2:00 pm and 7:00 pm and a gradual build-up from 7:00 pm to 5:00 am. The highest eBC_ff_ concentrations within the morning peak are attributed to the emissions resulting from intense vehicular activities during rush hours. The decrease in the eBC_ff_ concentration between 10:00 am and 2:00 pm could be explained by the enhanced unstable atmospheric conditions as a consequence of stronger solar radiation and winds. The afternoon peak and increasing eBC_ff_ concentrations until 5:00 am might be ascribed to two main factors: (i) emissions from heavy-duty diesel trucks (HDDTs), which are allowed to enter main arterial roads of the Dammam metropolitan area within the following time intervals: 3:00–5:00 pm and 10:00 pm–5:00 am, and (ii) stable nocturnal atmospheric conditions due to the decreased vertical thermal convection and weakened surface wind speeds.

The hourly cycle of eBC_ff_ during the LD phase showed lower concentrations within a narrower distribution in comparison with the Pre-LD and Post-LD stages. The significant and sharp peak between 5:00 am and 10:00 am observed during the Pre-LD period dramatically weakened, which might be ascribed to the imposed lockdown measures. The lowest mean eBC_ff_ concentrations ranging within 1.2 ± 0.7 and 1.1 ± 0.7 µg/m^3^ were noticed between 10:00 am and 2:00 pm. The eBC_ff_ concentrations gradually increased starting from 3:00 pm, reached the maximum mean value of 2.1 ± 1.8 µg/m^3^ at 3:00 am, and displayed a stepwise decline trend until 10:00 am. Shipping activities and HDDT movements continued within the allowed time intervals to supply essential products to the market and public in compliance with COVID-19 safety measures and regulations forced by the KSA. These emission sources and the shallower nocturnal atmospheric boundary layer were the main reasons for the gradual eBC_ff_ concentration build-up between 3:00 pm and 5:00 am during the LD stage.

The daily mean eBC_ff_ concentrations within the Post-LD period were 1.5- and 2.0-times higher than those measured at Pre-LD and LD phases, respectively. The highest eBC_ff_ concentrations varied between 3.1 ± 1.9 and 4.4 ± 2.7 µg/m^3^ within a distinct broad peak period from 5:00 pm to 5:00 am. In contrast, the time interval between 8:00 am and 3:00 pm indicated the lowest concentrations in the range of 2.2 ± 1.3 and 2.7 ± 2.3 µg/m^3^. The sharp morning peak observed during the Pre-LD phase was less pronounced within the Post-LD period, which could possibly be explained by the suspension of all schools and educational institutions, which drastically reduced the number of vehicles in rush-hour traffic. Boosted traffic activities from the supply chain and logistic disruptions because of the COVID-19 pandemic resulted in a surge in fossil fuel consumption. The lower mean wind speed and long-range transport might be linked to the increased eBC_ff_ concentrations during the Post-LD period compared to the Pre-LD period.

The daily mean eBC_bb_ concentrations throughout the Pre-LD, LD, and Post-LD phases were 0.31 ± 0.23, 0.27 ± 0.22, and 0.41 ± 0.26 µg/m^3^, respectively. The distributions of eBC_bb_ hourly cycles were quite similar to those of eBC_ff_ concentrations along the Pre-LD and LD periods. On the other hand, hourly variations of eBC_bb_ concentrations within the Post-LD phase demonstrated a particular bimodal distribution with four sharp peaks at 3:00 am (0.50 ± 0.34 µg/m^3^), 7:00 am (0.44 ± 0.27 µg/m^3^), noon (0.39 ± 0.32 µg/m^3^), and 8:00 pm (0.52 ± 0.27 µg/m^3^). The highest eBC_bb_ concentrations noticed within the sharp peak between 4:00 pm and 2:00 am might be due to emissions from biomass-burning stoves in restaurants and open-air barbecues under weak surface wind speeds and lower atmospheric boundary layer conditions.

### 3.2. Monthly Changes in BC Concentrations

**Figure 4:**
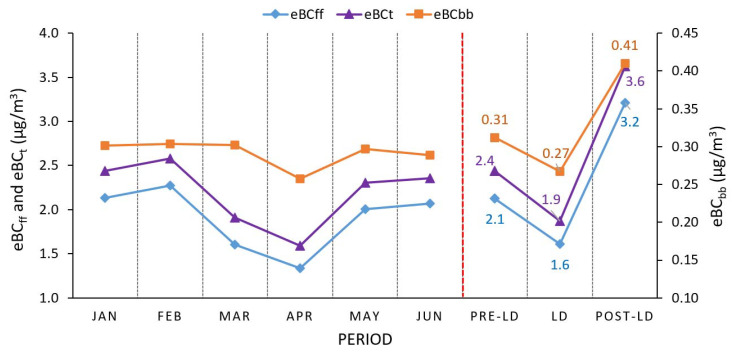
Monthly changes in equivalent black carbon (eBC) concentrations and their variations through the Pre-LD, LD, and Post-LD stages.

Monthly variations in eBC concentrations and their changes during Pre-LD, LD, and Post-LD phases are illustrated in Figure 4. Monthly changes in eBC_ff_ concentrations are more apparent than those of eBC_bb_ concentrations. The eBC_ff_ concentrations observed in January slightly changed in February by increasing from 2.1 ± 1.1 to 2.3 ± 0.9 µg/m^3^. Afterward, the mean eBC_ff_ concentration sharply decreased in March by 29.4%, which might presumably be attributed to the imposed measures to combat the COVID-19 pandemic. Compared to February, the change in eBC_ff_ concentrations was most evident in April as a 24-h LD was set nationwide between 6 April and 28 May. The lowest mean eBC_ff_ and eBC_bb_ concentrations were recorded as 1.3 ± 0.6 and 0.26 ± 0.07 µg/m^3^, respectively, in April. During the entire LD period, the public’s movement was restricted, except when accessing essential nearby facilities, such as food stores, pharmacies, and hospitals. The previous activities were allowable through obtaining an emergency permit between 6:00 am and 3:00 pm using the official Tawakkalna mobile application. Despite the full LD stringent measures, both eBC_ff_ and eBC_bb_ concentrations observed during May increased by 50.5 and 15.4%, respectively, compared to those measured in April. Besides, the mean eBC_t_ concentration in May was 11% lower than that recorded in February. The mean eBC_ff_ and eBC_bb_ concentrations during June were 2.1 ± 1.2 and 0.29 ± 0.12 µg/m^3^, respectively, and reverted the levels observed in January as the 24-h LD was partially lifted on 28 May.

Domestic heating, burning of crop residues, and forest fires have been reported as primary sources of BC_bb_ emissions, contributing significantly to the BC budget in other regions, such as Brazil [47], Canada [46], China [87], Finland [88], Greece [89], India [90], Italy [91], Spain [77], Turkey [41], and the USA [92]. However, wood-burning stoves in restaurants and open-air barbecues are the only eBC_bb_ sources within the study area and most parts of the KSA, since domestic heating is provided by air conditioners/electric heaters and farms/forested lands are very scarce. The study area’s biomass-burning characteristic mentioned herein is the main reason why the ratios of eBC_bb_ concentrations to eBC_t_ concentrations (BB_fraction_) were estimated as low as 0.16 ± 0.12, 0.17 ± 0.09, and 0.13 ± 0.06 throughout the Pre-LD, LD, and Post-LD stages, respectively. These results suggested that fossil fuel emissions dominated the ambient eBC_t_ budget over the study area by representing 84% of the eBC_t_ concentrations during the entire study period.

The mean eBC_bb_ and eBC_ff_ concentrations during the LD stage dropped by 14.2% and 24.2% and then increased by 31.7% and 51.4%, respectively, after the release of LD measures in reference to the Pre-LD phase. This unexpected increase, especially in eBC_ff_ concentrations, after lifting of the LD was most probably derived from (i) exertions for restoring supply-chain losses, such as elevated fossil fuel consumption in transportation and industrial activities, (ii) escalated construction activities in the vicinity of the study area, (iii) more stable atmospheric conditions due to lower wind speed compared to the Post-LD phase, and (iv) long-range transport of BC particles from other regions to the receptor site.

### 3.3. Effect of Meteorology and Local Sources

A bivariate correlation analysis was applied to hourly mean values of both meteorological parameters and eBC fractions for the whole study period in order to reveal the effects of local meteorology on ambient eBC_ff_, eBC_bb_, and eBC_t_ concentrations. The Pearson correlation coefficients between each parameter are shown in Table 2. Moderate negative correlations were found between wind speed and eBC_ff_ (r = −0.445), eBC_bb_ (−0.375), and eBC_t_ (−0.435) concentrations, suggesting that BC particles were more dispersed under higher wind speeds or that they accumulated at lower wind speeds, as also reported by other studies [33,88,93]. The effect of wind direction on eBC_t_ concentrations was further evaluated using bivariate polar plots because of the polar characteristic of wind direction data (between 0 and 360°). Other meteorological parameters did not yield any statistically significant correlations with the measured eBC concentrations. eBC_t_ concentrations resulted in strong positive correlations with eBC_ff_ (0.996) and eBC_bb_ concentrations (0.634), implying that the eBC_t_ composition was mostly represented by the eBC_ff_ fraction during the study period.

**Figure 5:**
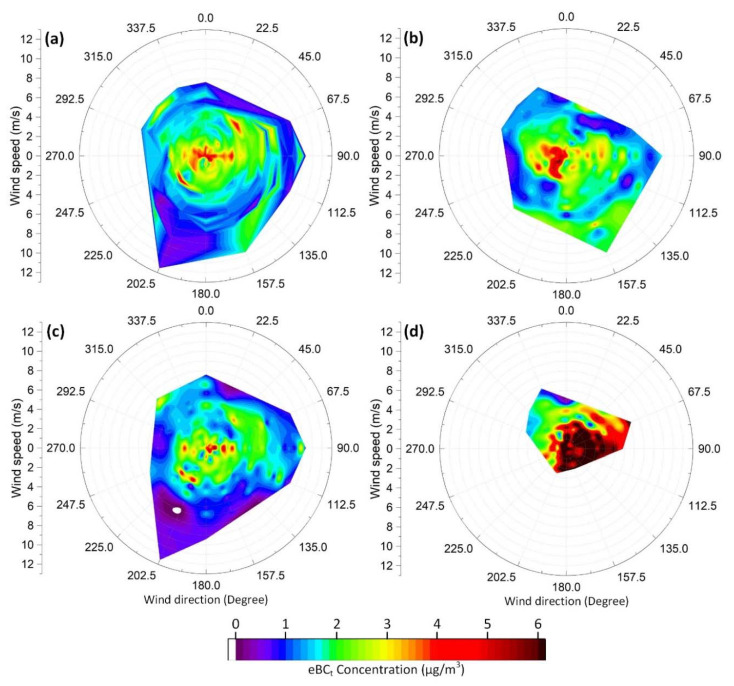
Bivariate polar plots demonstrating the effect of wind direction and wind velocity on measured eBC_t_ concentrations during: (**a**) the whole study, (**b**) Pre-LD, (**c**) LD, and (**d**) Post-LD periods.

Bivariate polar plots of concentrations were graphed to find out the conjunct effect of wind velocity and wind direction on hourly mean eBC_t_ concentrations and potential source locations of eBC_t_ emissions affecting the receptor site (Figure 5). It can be inferred from Figure 5a that low wind speeds (<2 m/s) were mostly accompanied by high eBC_t_ concentrations (>4 µg/m^3^), revealing that local sources mainly dominated the observed high concentrations during the entire study period. On the other hand, moderate (2–4 µg/m^3^) and high (>4 µg/m^3^) concentrations were also brought on by high-speed winds blowing from varying directions to the monitoring site, implying the potential contributions of regional and long-range sources to the measured concentrations. The Pearson correlation coefficients between the wind speed and eBC_t_ concentrations were found to be −0.674, −0.271, and −0.515 for the Pre-LD, LD, and Post-LD stages, respectively. These correlation levels could suggest that local sources’ effects were more evident in the measured concentrations at the Pre-LD and Post-LD stages.

The low-speed winds (<4 m/s) blowing from westerly sectors most likely transported the fossil-fuel-combustion plumes originating from nearby emission spots, such as the King Abdulaziz Sea Port, King Abdulaziz and King Fahd roads, and industrial area #1, which resulted in moderate-to-high concentrations at the monitoring site throughout the Pre-LD, LD, and Post-LD phases, as seen in Figure 5b–d, respectively. During the Pre-LD period, the easterly sectors demonstrated moderate concentrations at variable wind speeds. Contrary to the Pre-LD stage, the effect of westerly winds on eBC_t_ concentrations was less pronounced, which could possibly be due to dramatically reduced traffic volume during the LD. The highest concentrations at low wind speeds and moderate concentrations at variable wind speeds were, instead, associated with the LD’s easterly directions. The emissions from cargo ships docking to and hailing from the King Abdulaziz Sea Port, land reclamation activities, and transboundary pollution transport might be the main reasons giving rise to the elevated eBC_t_ concentrations at the receptor site since there are no other known local emission sources on the east section of the monitoring site. Throughout the Post-LD phase, eBC_t_ concentrations showed a distinct distribution on the bivariate polar plot, depicted in Figure 5d. It was observed that intense land reclamation construction activities had started at the sector between NE and SE directions with respect to the monitoring location during the Post-LD period. Diesel-fuel-combustion plumes of heavy-duty excavators and trucks operating on the nearby construction site were carried to the monitoring site by low-speed winds (<3 m/s) and, thus, led to the highest concentrations (>8 µg/m^3^) in the presence of atmospheric inversion conditions.

### 3.4. Identification of Long-Range Sources

#### 3.4.1. Cluster Analysis

**Figure 6:**
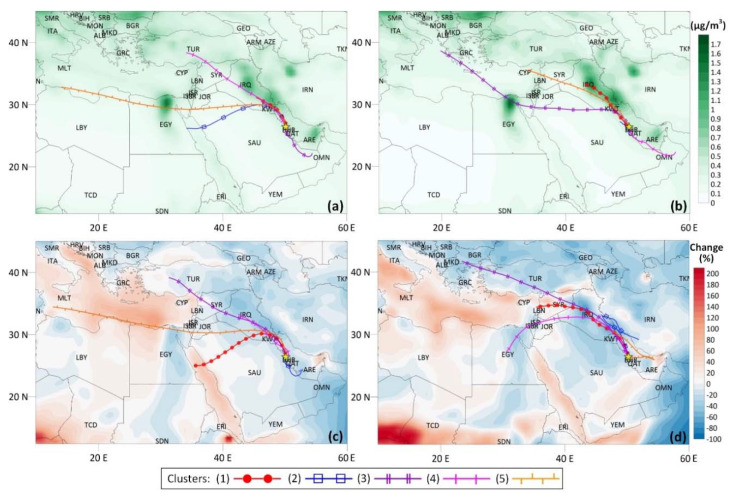
Clusters of three-day backward trajectories superimposed on satellite-based total BC (BCt) surface data along (**a**) the entire study and (**b**) Pre-LD periods with mass concentrations; (**c**) LD and (**d**) Post-LD periods with percent change in mass concentrations as compared to the Pre-LD period.

The cluster analysis model grouped backward air mass trajectories to the whole study, Pre-LD, LD, and Post-LD periods into five distinct clusters as an optimum number of clusters according to the objective percentage change criterion applied for the total spatial variance of each cluster solution. The frequency of backward trajectories of each cluster in all clusters and the mean value of measured eBC_t_ concentrations assigned to backward trajectories in each cluster are given in Table 3. The mean trajectories of clusters were superimposed on the satellite-based BC surface mass concentration data to visualize the BC emissions spots that might enrich the air masses traveling over them and eventually reaching the receptor site (Figure 6).

Figure 6a demonstrates that air mass trajectories traversing the NNE zone of Egypt, the United Arab Emirates, and central Iraq and the SE section of Iraq with borders with Kuwait and Iran were presumably highly enriched with BC since these areas yielded the highest BC emissions according to the satellite observations. The mean trajectories of clusters reaching the study area from the NNW section have different origins, but they followed a familiar route during the last 12 h of their travel time. The zone, including the SE region of Iraq and Kuwait and the NE section of the Arabian Gulf, commonly passed over by the corresponding air masses, has a significant potential to contribute to the elevated eBC concentrations measured at the monitoring site. Anil et al. [82] also classified this zone as a hot spot emitting high amounts of SO_2_, NO_2_, and PM_10_ pollutants in their recent study investigating transport pathways on the chemical composition of wet deposition over the Dammam area.

Satellite observations show higher mean BC concentration distributions over the mapped domain for the Pre-LD period as compared with the LD and Post-LD periods, except over the Mediterranean Sea, Red Sea, and most parts of Africa (Figure 6b–d). During the Pre-LD period, clusters 1 and 2 classifying 56% of all air mass trajectories represented the highest mean eBC concentrations of 2.7 and 2.8 µg/m^3^, respectively, among other clusters. Clusters 1 and 2, including slow-moving air masses originated from central Iraq and the NE part of the KSA, were enhanced with long-range and regional BC emissions and arrived at the receptor site from NW and S directions, respectively. The trajectories in cluster 2 could be ascribed to regionally polluted air masses by fossil fuel burning emissions as they crossed over heavily industrialized areas of Jubail, Ras Tanura, and Qatif cities, leading to high eBC concentrations in the study area.

The lower mean BC concentration distributions had been experienced over most of the countries within the studied domain, which might be explained by the LD countermeasures imposed in those countries (Figure 6c). The air mass trajectories with the highest mean eBC concentrations of 2.3 and 2.0 µg/m^3^ were associated with clusters 1 and 2, describing 31.1% and 27.8% of all trajectories, respectively, throughout the LD phase. Cluster 1, deriving from the Red Sea coast of Egypt, traveled over the northern part of the KSA, enriched with BC emissions over the SE region of Iraq, Kuwait, and the eastern coast of the KSA, and contributed to the receptor’s BC budget. Cluster 2, representing slow-moving and low-altitude (850 m AGL) air masses, was more effectively polluted by the industrial and urban emissions along the pathway through the United Arab Emirates, Buqayq, and Al Hofuf cities of the KSA, and eventually arrived at the study area from the SSW section.

Satellite observations indicated slightly higher mean BC concentration dispersions during the LD period compared to the Pre-LD period (Figure 6d). Cluster 1, accounting for 50% of the back trajectories, originated from Lebanon, traversed Syria, Iraq, and Kuwait, and eventually brought moderately enriched air masses to the receptor site, resulting in the measured mean eBC concentration of 3.2 µg/m^3^. Clusters 2 and 5, representing the lowest number of air mass trajectories, induced the highest observed mean concentrations of 6.2 and 7.0 µg/m^3^, respectively. These extreme concentrations could be attributed to slow and low-altitude (820 m AGL) flowing characteristics of the clusters that are intensely loaded with anthropogenic emissions arising from Iran’s western border, the eastern region of Iraq, Kuwait, United Arab Emirates, and Bahrain during their residence times.

#### 3.4.2. Concentration-Weighted Trajectory Analysis

**Figure 7:**
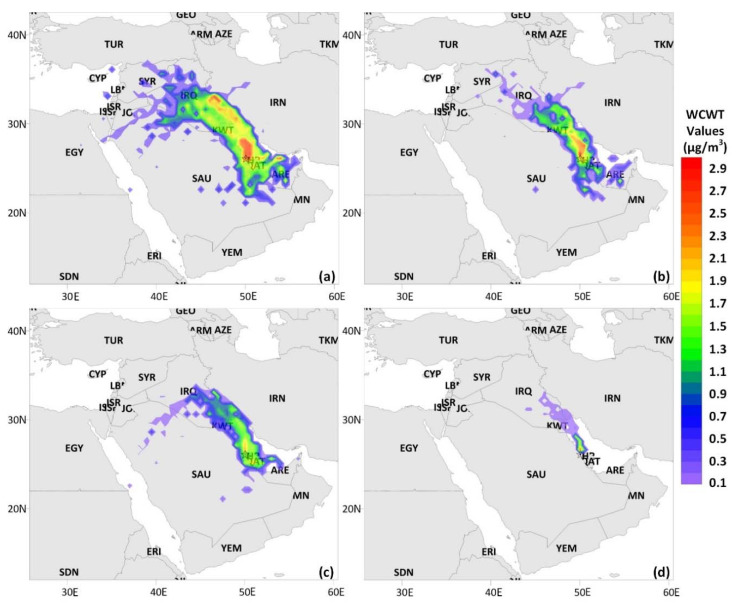
Concentration-weighted trajectory (CWT) analysis results for: (**a**) the whole study, (**b**) Pre-LD, (**c**) LD, and (**d**) Post-LD stages.

The CWT analysis was further applied in order to quantitatively determine the relative contributions of regional and long-range source regions to the ambient eBC_t_ concentrations in the study area, since the capability of the cluster analysis is quite limited in delineating the effects of potential source regions at the receptor site in detail. The CWT analysis results of the entire study, Pre-LD, LD, and Post-LD periods are visualized in Figure 7, where the weighted concentration (WCWT, µg/m^3^) calculated for each cell (0.5° × 0.5°) demonstrates the contribution level of a potential source location at the receptor site.

During the entire study period, long-range source locations with WCWT values > 1 µg/m^3^, including the central and southeastern section of Iraq, Iran’s western border with Iraq, Kuwait, distributed zones in the Arabian Gulf, and the United Arab Emirates, were associated with moderate to high eBC concentrations observed at the receptor site (Figure 7a). The impacts of long-range source zones mentioned herein were also recently reported for the BC levels over Riyadh and Dammam’s rainwater chemical compositions [52,83]. In addition, it is evident from Figure 7a that the effects of regional source locations, such as the Arabian Gulf coastline of the KSA, Bahrain, and Qatar, with moderate to high WCWT values on the observed eBC levels were substantial. These medium- and long-range potential source zones were, thus, coherent with the idea that the advections of emissions from fossil fuel combustion at factories and power plants, gas flaring at oil refineries, transportation activities at densely populated urban cores, and heavy maritime traffic of oil tankers and cargo ships on the Arabian Gulf contributed to the BC budget over Dammam’s atmosphere.

The advection of air masses from long-range potential source zones, such as Iraq, Iran, Kuwait, and the United Arab Emirates, with WCWT values between 1 and 2 µg/m^3^, moderately contributed to the observed eBC concentrations in the study area through the Pre-LD phase (Figure 7b). The highest concentrations measured during the Pre-LD period were accompanied by regional source emissions from heavy industrial activities in Jubail city, oil production fields and petrochemical industries in Qatif city, and intense transportation activities on the Arabian Gulf, further adding to high local BC emissions within the Dammam area. The WCWT values remarkably decreased during the LD period. This decrease could be possibly due to the reduced traffic and industrial emissions in response to the applied LD measures by many countries (Figure 7c). Within this period, contributions from regional source locations, including Qatar, Bahrain, and Jubail city, to the observed eBC levels were significant. The WCWT values indicated a relatively narrow distribution in the post-LD stage (Figure 7d). The high eBC concentrations measured at the monitoring station were mainly of local origin. They could mostly be attributed to the industrial emissions from Ras Tanura city, located 40 km to the NNW direction, and local emissions in the vicinity of the study area, as discussed in Section “3.3. Effect of meteorology and local sources”.

### 3.5. Comparison with COVID-19 Literature

Worldwide ground-level BC concentrations were compared with the BC data observed in the study area before and during the LD, as given in Table 4. Before the LD period, the highest BC levels were reported in populated urban cores, such as Suzhou and Chongqing of China and Milan of Italy, where local emissions from traffic, industrial, household heating, and cooking activities have been significantly contributing the ambient BC budget over those regions. The mean BC concentration measured in Dammam was higher than those in Bhubaneswar/India and Massachusetts/USA, most likely due to more extensive industrial and transportation activities in the study area. The BC levels indicated decreasing trends with varying rates for all compared sites in line with COVID-19 LD measures. The potential effect of LD countermeasures on BC emissions was more pronounced in Milan/Italy, where the highest decrease ratio of 71% was noticed. The mean BC reduction rate observed in Dammam is one of the lowest among others. This result could be attributed to a wide range of emission sources with varying speeds, intrinsic atmospheric stability, specific arid climate conditions of the Dammam area, and long-range sources.

## 4. Conclusions

This research presents the first attempt to report ground-level measurements of eBC mass concentrations for the Eastern Province of Saudi Arabia, particularly for the Dammam metropolitan area. Continuous monitoring of hourly eBC concentrations was initiated at a university campus’ premises between 8 January and 2 July 2020. The eBC_ff_ and eBC_bb_ fractions in total eBC concentrations were estimated as 84% and 16%, respectively, by using the aethalometer data during the entire study period. Hourly variations of eBC_ff_ and eBC_bb_ levels during Pre-LD, LD, and Post-LD periods indicated distinct bimodal distributions. Before the implementation of COVID-19 LD measures, the highest eBC concentrations within the significant and sharp morning peak (5:00–10:00 am), the lowest concentrations between 10:00 am and 2:00 pm, and the minor peak (2:00 pm and 7:00 pm) along with the gradual concentration build-up from 7:00 pm to 5:00 am were attributed to (i) intense vehicular activities during morning rush hours, (ii) enhanced atmospheric instability around midday, and (iii) emissions from HDDTs under stable nocturnal atmospheric conditions, respectively. The mean eBC_bb_, eBC_ff_, and eBC_t_ concentrations during the LD decreased by 14%, 24%, and 23%, respectively. The reductions in eBC levels within the LD period could be possibly attributed to the strict restrictions on public movement and activities for preventing the spread of COVID-19, further adding to changes in atmospheric conditions and effects of long-range transport. The results of the bivariate correlation analysis and polar plots of concentrations revealed obvious influences of local fossil fuel burning emissions and atmospheric conditions on the observed eBC levels. Besides, distinct regional and long-range source regions contributing to the measured concentrations at the receptor site were identified for the Pre-LD, LD, and Post-LD periods by using a cluster analysis of backward air mass trajectories and CWT analysis. Long-range potential source locations, including Iraq, Kuwait, Iran, distributed zones in the Arabian Gulf, and the United Arab Emirates, and regional source areas, such as the Arabian Gulf coastline of KSA, Bahrain, and Qatar, were associated with moderate to high concentrations observed at the receptor site. The nationwide COVID-19 LD has procured a unique chance for the atmospheric researchers, policymakers, and administrative bodies to assess the effects of emission reductions on the ambient air quality and to rethink the current and future air pollution mitigation strategies.

## Figures and Tables

**Table 1:** Descriptive statistics of the measured parameters during Pre-LD, LD, and Post-LD.

Parameters	Period	Mean	Median	Mode	S.D.	Min.	Max.	Percentiles		
								25th	50th	75th
Temperature (°C)	Pre-LD	18.8	18.9	20.4	3.65	7.30	29.6	16.4	18.9	21.3
	LD	29.9	29.6	28.6	5.42	15.8	45.6	25.7	29.6	34.0
	Post-LD	35.3	34.8	33.6 ^a^	3.03	29.6	44.3	33.2	34.8	37.4
Humidity (%)	Pre-LD	66.2	67.0	64.0	14.6	27.0	97.0	56.0	67.0	78.0
	LD	50.2	49.0	38.0	18.6	12.0	92.0	36.0	49.0	66.0
	Post-LD	42.5	40.0	46.0	18.7	12.0	88.0	28.3	40.0	54.0
Wind Speed (m/s)	Pre-LD	3.2	3.1	3.6	1.8	0.0	10.7	1.8	3.1	4.5
	LD	3.3	3.1	3.1	1.8	0.0	12.5	1.8	3.1	4.5
	Post-LD	2.8	2.7	1.3 ^a^	1.5	0.0	7.2	1.8	2.7	4.0
Pressure (mbar)	Pre-LD	1019	1019	1020	4.4	1007	1032	1017	1019	1022
	LD	1008	1009	1011	4.8	997	1020	1005	1009	1012
	Post-LD	1000	1000	1001	1.7	996	1003	999	1000	1002
Solar Radiation (W/m^2^)	Pre-LD	188	0.0	0.0	265	0.0	871	0.0	0.0	376
	LD	246	40.0	0.0	313	0.0	953	0.0	40.0	480
	Post-LD	270	48.0	0.0	323	0.0	895	0.0	48.0	538
eBC_bb_ (µg/m^3^)	Pre-LD	0.31	0.27	0.20	0.23	0.01	3.6	0.17	0.27	0.39
	LD	0.27	0.22	0.14 ^a^	0.22	0.02	3.6	0.15	0.22	0.33
	Post-LD	0.41	0.35	1.1	0.27	0.06	1.5	0.22	0.35	0.53
eBC_ff_ (µg/m^3^)	Pre-LD	2.1	1.8	1.5	1.5	0.12	11.1	1.1	1.8	2.7
	LD	1.6	1.2	0.79 ^a^	1.4	0.12	13.4	0.74	1.2	2.0
	Post-LD	3.2	2.4	9.9	2.3	0.53	12.8	1.3	2.4	4.8
eBC_t_ (µg/m^3^)	Pre-LD	2.4	2.1	1.8	1.6	0.33	12.3	1.4	2.1	3.0
	LD	1.9	1.4	0.93 ^a^	1.5	0.25	14.1	0.91	1.4	2.3
	Post-LD	3.6	2.7	11.0	2.5	0.67	14.2	1.5	2.7	5.3

^a^ Multiple modes exist. The smallest value is shown.

**Table 2:** Statistical correlations between black carbon fractions and meteorological parameters.

Parameters	Temperature	Humidity	Wind Speed	Pressure	Rainfall	Solar Radiation	eBC_ff_	eBC_bb_
Humidity	−0.672 *							
Wind speed	0.019	−0.057						
Pressure	−0.952 *	0.562 *	−0.066					
Rainfall	−0.112	0.142	0.015	0.069				
Solar radiation	0.631 *	−0.575 *	−0.147	−0.548 *	−0.277 *			
eBC_ff_	0.004	0.253	−0.445 *	−0.014	−0.075	0.051		
eBC_bb_	0.014	0.202	−0.375 *	−0.066	−0.080	−0.018	0.562 *	
eBC_t_	0.005	0.259	−0.435 *	−0.021	−0.078	0.046	0.996 *	0.634 *

* Correlation is significant at the 0.01 level (two-tailed).

**Table 3:** Cluster statistics of backward air mass trajectories.

Period	Parameters	Clusters				
		1	2	3	4	5
All	Frequency (%)	31.1	20.3	19.2	23.7	5.7
	eBC_t_ (µg/m^3^)	2.7	2.2	2.1	1.9	1.6
Pre-Lockdown	Frequency (%)	28.0	28.0	8.0	14.7	21.3
	eBC_t_ (µg/m^3^)	2.7	2.8	1.5	2.0	2.3
Lockdown	Frequency (%)	31.1	27.8	15.6	17.8	7.8
	eBC_t_ (µg/m^3^)	2.3	2.0	1.2	1.7	1.5
Post-Lockdown	Frequency (%)	50.0	8.3	16.7	16.7	8.3
	eBC_t_ (µg/m^3^)	3.2	6.2	2.1	3.6	7.0

**Table 4:** BC levels and changes in different regions before and during COVID-19 lockdowns.

Location	LD Period	BC Concentration (µg/m^3^)		Reduction (%)	Reference
		Pre-LD	LD		
Massachusetts/USA	24 March–8 June	0.5–0.7	0.34–0.42	22–46	[66]
Milan/Italy	9 March–5 April	3.9	1.1	71	[67]
Chongqing/China	24 January–24 March	5.1	2.9	43	[68]
Suzhou/China	27 January–31 March	3.2	1.5	53	[69]
Bhubaneswar/India	22 March–1 June	1.8	0.96	47	[70]
Dammam/KSA	23 March–20 June	2.4 ± 1.6	1.9 ± 1.5	23	Current Study

## References

[1] Wu J., Lu J., Min X., Zhang Z. (2018). Distribution and health risks of aerosol black carbon in a representative city of the Qinghai-Tibet Plateau. Environ. Sci. Pollut. Res..

[2] Li Y., Henze D.K., Jack D., Henderson B.H., Kinney P.L. (2016). Assessing public health burden associated with exposure to ambient black carbon in the United States. Sci. Total Environ..

[3] Segersson D., Eneroth K., Gidhagen L., Johansson C., Omstedt G., Nylén A.E., Forsberg B. (2017). Health impact of PM10, PM2.5 and black carbon exposure due to different source sectors in Stockholm, Gothenburg and Umea, Sweden. Int. J. Environ. Res. Public Health.

[4] Tecer L.H., Alagha O., Karaca F., Tuncel G., Eldes N. (2008). Particulate matter (PM2.5, PM10-2.5, and PM 10) and children’s hospital admissions for asthma and respiratory diseases: A bidirectional case-crossover study. J. Toxicol. Environ. Health Part A Curr. Issues.

[5] Gardiner K. (2001). Respiratory health effects from exposure to carbon black: Results of the phase 2 and 3 cross sectional studies in the European carbon black manufacturing industry. Occup. Environ. Med..

[6] Gu Y., Zhang W., Yang Y., Wang C., Streets D.G., Yim S.H.L. (2020). Assessing outdoor air quality and public health impact attributable to residential black carbon emissions in rural China. Resour. Conserv. Recycl..

[7] Gardiner K., Trethowan N.W., Harrington J.M., Rossiter C.E., Calvert I.A. (1993). Respiratory health effects of carbon black: A survey of European carbon black workers. Br. J. Ind. Med..

[8] Bové H., Bongaerts E., Slenders E., Bijnens E.M., Saenen N.D., Gyselaers W., Van Eyken P., Plusquin M., Roeffaers M.B.J., Ameloot M. (2019). Ambient black carbon particles reach the fetal side of human placenta. Nat. Commun..

[9] Tang C.-S., Chuang K.-J., Chang T.-Y., Chuang H.-C., Chen L.-H., Lung S.-C.C., Chang L.-T. (2019). Effects of Personal Exposures to Micro- and Nano-Particulate Matter, Black Carbon, Particle-Bound Polycyclic Aromatic Hydrocarbons, and Carbon Monoxide on Heart Rate Variability in a Panel of Healthy Older Subjects. Int. J. Environ. Res. Public Health.

[10] Choi S., Park J.-H., Kim S.-Y., Kwak H., Kim D., Lee K.-H., Park D.-U. (2019). Characteristics of PM2.5 and Black Carbon Exposure Among Subway Workers. Int. J. Environ. Res. Public Health.

[11] Olstrup H., Johansson C., Forsberg B., Åström C. (2019). Association between Mortality and Short-Term Exposure to Particles, Ozone and Nitrogen Dioxide in Stockholm, Sweden. Int. J. Environ. Res. Public Health.

[12] Karaca F., Anil I., Alagha O. (2009). Long-range potential source contributions of episodic aerosol events to PM10 profile of a megacity. Atmos. Environ..

[13] Anıl I., Golcuk K., Karaca F. (2014). ATR-FTIR Spectroscopic Study of Functional Groups in Aerosols: The Contribution of a Saharan Dust Transport to Urban Atmosphere in Istanbul, Turkey. Water Air Soil Pollut..

[14] Cape J.N., Coyle M., Dumitrean P. (2012). The atmospheric lifetime of black carbon. Atmos. Environ..

[15] Wang B., Chen H., Yik X., Chan L., Oliver B.G. (2020). Is there an association between the level of ambient air pollution and COVID-19?. J. Physiol. Lung Cell Mol. Physiol..

[16] Singh S., Tiwari S., Hopke P.K., Zhou C., Turner J.R., Panicker A.S., Singh P.K. (2018). Ambient black carbon particulate matter in the coal region of Dhanbad, India. Sci. Total Environ..

[17] Chandra S., Kulshrestha M.J., Singh R., Singh N. (2017). Chemical characteristics of trace metals in PM10 and their concentrated weighted trajectory analysis at Central Delhi, India. J. Environ. Sci..

[18] Bansal O., Singh A., Singh D. (2019). Characteristics of Black Carbon aerosols over Patiala Northwestern part of the IGP: Source apportionment using cluster and CWT analysis. Atmos. Pollut. Res..

[19] Guan Q., Yang Y., Luo H., Zhao R., Pan N., Lin J., Yang L. (2019). Transport pathways of PM10 during the spring in northwest China and its characteristics of potential dust sources. J. Clean. Prod..

[20] Hsu Y.K., Holsen T.M., Hopke P.K. (2003). Comparison of hybrid receptor models to locate PCB sources in Chicago. Atmos. Environ..

[21] Seibert P., Kromp-Kolb H., Kasper A., Kalina M., Puxbaum H., Jost D.T., Schwikowski M., Baltensperger U. (1998). Transport of polluted boundary layer air from the PO valley to high- alpine sites. Atmos. Environ..

[22] Maritz P., Beukes J.P., van Zyl P.G., Conradie E.H., Liousse C., Galy-Lacaux C., Castéra P., Ramandh A., Mkhatshwa G., Venter A.D. (2015). Spatial and temporal assessment of organic and black carbon at four sites in the interior of South Africa. Clean Air J..

[23] Doumbia E.H.T., Liousse C., Galy-Lacaux C., Ndiaye S.A., Diop B., Ouafo M., Assamoi E.M., Gardrat E., Castera P., Rosset R. (2012). Real time black carbon measurements in West and Central Africa urban sites. Atmos. Environ..

[24] Kuik F., Lauer A., Beukes J.P., Van Zyl P.G., Josipovic M., Vakkari V., Laakso L., Feig G.T. (2015). The anthropogenic contribution to atmospheric black carbon concentrations in southern Africa: A WRF-Chem modeling study. Atmos. Chem. Phys..

[25] Altstädter B., Deetz K., Vogel B., Babić K., Dione C., Pacifico F., Jambert C., Ebus F., Bärfuss K., Pätzold F. (2020). The vertical variability of black carbon observed in the atmospheric boundary layer during DACCIWA. Atmos. Chem. Phys..

[26] Khan A.L., Klein A.G., Katich J.M., Xian P. (2019). Local Emissions and Regional Wildfires Influence Refractory Black Carbon Observations Near Palmer Station, Antarctica. Front. Earth Sci..

[27] Bisiaux M.M., Edwards R., McConnell J.R., Curran M.A.J., Van Ommen T.D., Smith A.M., Neumann T.A., Pasteris D.R., Penner J.E., Taylor K. (2012). Changes in black carbon deposition to Antarctica from two high-resolution ice core records, 1850–2000 AD. Atmos. Chem. Phys..

[28] Arienzo M.M., McConnell J.R., Murphy L.N., Chellman N., Das S., Kipfstuhl S., Mulvaney R. (2017). Holocene black carbon in Antarctica paralleled Southern Hemisphere climate. J. Geophys. Res. Atmos..

[29] Chaubey J.P., Moorthy K.K., Babu S.S., Nair V.S., Tiwari A. (2010). Black carbon aerosols over coastal Antarctica and its scavenging by snow during the Southern Hemispheric summer. J. Geophys. Res..

[30] Hansen A.D.A., Lowenthal D.H., Chow J.C., Watson J.G. (2001). Black Carbon Aerosol at McMurdo Station, Antarctica. J. Air Waste Manage. Assoc..

[31] Babu S.S., Chaubey J.P., Krishna Moorthy K., Gogoi M.M., Kompalli S.K., Sreekanth V., Bagare S.P., Bhatt B.C., Gaur V.K., Prabhu T.P. (2011). High altitude (∼4520 m amsl) measurements of black carbon aerosols over western trans-Himalayas: Seasonal heterogeneity and source apportionment. J. Geophys. Res. Atmos..

[32] Girach I.A., Nair V.S., Babu S.S., Nair P.R. (2014). Black carbon and carbon monoxide over Bay of Bengal during W_ICARB: Source characteristics. Atmos. Environ..

[33] Tiwari S., Srivastava A.K., Bisht D.S., Parmita P., Srivastava M.K., Attri S.D. (2013). Diurnal and seasonal variations of black carbon and PM2.5 over New Delhi, India: Influence of meteorology. Atmos. Res..

[34] Cheng Y., He K.B., Du Z.Y., Engling G., Liu J.M., Ma Y.L., Zheng M., Weber R.J. (2016). The characteristics of brown carbon aerosol during winter in Beijing. Atmos. Environ..

[35] Duc H.N., Shingles K., White S., Salter D., Chang L.T.-C., Gunashanhar G., Riley M., Trieu T., Dutt U., Azzi M. (2020). Spatial-Temporal Pattern of Black Carbon (BC) Emission from Biomass Burning and Anthropogenic Sources in New South Wales and the Greater Metropolitan Region of Sydney, Australia. Atmosphere.

[36] Surawski N.C., Sullivan A.L., Roxburgh S.H., Polglase P.J. (2016). Estimates of greenhouse gas and black carbon emissions from a major Australian wildfire with high spatiotemporal resolution. J. Geophys. Res. Atmos..

[37] Weingartner E., Saathoff H., Schnaiter M., Streit N., Bitnar B., Baltensperger U. (2003). Absorption of light by soot particles: Determination of the absorption coefficient by means of aethalometers. J. Aerosol Sci..

[38] Olivares G., Ström J., Johansson C., Gidhagen L. (2008). Estimates of Black Carbon and Size-Resolved Particle Number Emission Factors from Residential Wood Burning Based on Ambient Monitoring and Model Simulations. J. Air Waste Manage. Assoc..

[39] Ferrero L., Mocnik G., Ferrini B.S., Perrone M.G., Sangiorgi G., Bolzacchini E. (2011). Vertical profiles of aerosol absorption coefficient from micro-Aethalometer data and Mie calculation over Milan. Sci. Total Environ..

[40] Zanatta M., Gysel M., Bukowiecki N., Müller T., Weingartner E., Areskoug H., Fiebig M., Yttri K.E., Mihalopoulos N., Kouvarakis G. (2016). A European aerosol phenomenology-5: Climatology of black carbon optical properties at 9 regional background sites across Europe. Atmos. Environ..

[41] Kuzu S.L., Yavuz E., Akyüz E., Saral A., Akkoyunlu B.O., Özdemir H., Demir G., Ünal A. (2020). Black carbon and size-segregated elemental carbon, organic carbon compositions in a megacity: A case study for Istanbul. Air Qual. Atmos. Heal..

[42] Gundel L.A., Dod R.L., Rosen H., Novakov T. (1984). The relationship between optical attenuation and black carbon concentration for ambient and source particles. Sci. Total Environ..

[43] Huang L., Gong S.L., Sharma S., Lavoué D., Jia C.Q. (2010). A trajectory analysis of atmospheric transport of black carbon aerosols to Canadian high Arctic in winter and spring (1990–2005). Atmos. Chem. Phys..

[44] Ahmed T., Dutkiewicz V.A., Khan A.J., Husain L. (2014). Long term trends in Black Carbon Concentrations in the Northeastern United States. Atmos. Res..

[45] Devi J.J., Bergin M.H., Mckenzie M., Schauer J.J., Weber R.J. (2016). Contribution of particulate brown carbon to light absorption in the rural and urban Southeast US. Atmos. Environ..

[46] Healy R.M., Sofowote U., Su Y., Debosz J., Noble M., Jeong C.H., Wang J.M., Hilker N., Evans G.J., Doerksen G. (2017). Ambient measurements and source apportionment of fossil fuel and biomass burning black carbon in Ontario. Atmos. Environ..

[47] Diaz Resquin M., Santágata D., Gallardo L., Gómez D., Rössler C., Dawidowski L. (2018). Local and remote black carbon sources in the Metropolitan Area of Buenos Aires. Atmos. Environ..

[48] De Miranda R.M., Perez-Martinez P.J., de Fatima Andrade M., Ribeiro F.N.D. (2019). Relationship between black carbon (BC) and heavy traffic in São Paulo, Brazil. Transp. Res. Part D Transp. Environ..

[49] Morales Betancourt R., Galvis B., Balachandran S., Ramos-Bonilla J.P., Sarmiento O.L., Gallo-Murcia S.M., Contreras Y. (2017). Exposure to fine particulate, black carbon, and particle number concentration in transportation microenvironments. Atmos. Environ..

[50] Lihavainen H., Alghamdi M.A., Hyvärinen A.-P., Hussein T., Aaltonen V., Abdelmaksoud A.S., Al-Jeelani H., Almazroui M., Almehmadi F.M., Al Zawad F.M. (2016). Aerosols physical properties at Hada Al Sham, western Saudi Arabia. Atmos. Environ..

[51] Nayebare S.R., Aburizaiza O.S., Siddique A., Carpenter D.O., Hussain M.M., Zeb J., Aburiziza A.J., Khwaja H.A. (2018). Ambient air quality in the holy city of Makkah: A source apportionment with elemental enrichment factors (EFs) and factor analysis (PMF). Environ. Pollut..

[52] Bian Q., Alharbi B., Shareef M.M., Husain T., Pasha M.J., Atwood S.A., Kreidenweis S.M. (2018). Sources of PM2.5 carbonaceous aerosol in Riyadh, Saudi Arabia. Atmos. Chem. Phys..

[53] WHO Coronavirus Disease (COVID-19). https://www.who.int/emergencies/diseases/novel-coronavirus-2019.

[54] Bai Y., Zhou Y., Alatalo J.M., Hughes A.C. (2020). Changes in Air Quality during the First-Level Response to the Covid-19 Pandemic in Shanghai Municipality, China. Sustainability.

[55] Vultaggio M., Varrica D., Alaimo M.G. (2020). Impact on Air Quality of the COVID-19 Lockdown in the Urban Area of Palermo (Italy). Int. J. Environ. Res. Public Health.

[56] Ghosh S., Das A., Hembram T.K., Saha S., Pradhan B., Alamri A.M. (2020). Impact of COVID-19 Induced Lockdown on Environmental Quality in Four Indian Megacities Using Landsat 8 OLI and TIRS-Derived Data and Mamdani Fuzzy Logic Modelling Approach. Sustainability.

[57] Cheval S., Mihai Adamescu C., Georgiadis T., Herrnegger M., Piticar A., Legates D.R. (2020). Observed and Potential Impacts of the COVID-19 Pandemic on the Environment. Int. J. Environ. Res. Public Health.

[58] Anil I., Alagha O. (2020). The impact of COVID-19 lockdown on the air quality of Eastern Province, Saudi Arabia. Air Qual. Atmos. Heal..

[59] Broomandi P., Karaca F., Nikfal A., Jahanbakhshi A., Tamjidi M., Kim J.R. (2020). Impact of COVID-19 Event on the Air Quality in Iran. Aerosol Air Qual. Res..

[60] Donzelli G., Cioni L., Cancellieri M., Llopis Morales A., Morales Suárez-Varela M.M. (2020). The Effect of the Covid-19 Lockdown on Air Quality in Three Italian Medium-Sized Cities. Atmosphere.

[61] Seo J.H., Jeon H.W., Sung U.J., Sohn J.-R. (2020). Impact of the COVID-19 Outbreak on Air Quality in Korea. Atmosphere.

[62] Şahin Ü.A. (2020). The Effects of COVID-19 Measures on Air Pollutant Concentrations at Urban and Traffic Sites in Istanbul. Aerosol Air Qual. Res..

[63] Rossi R., Ceccato R., Gastaldi M. (2020). Effect of Road Traffic on Air Pollution. Experimental Evidence from COVID-19 Lockdown. Sustainability.

[64] Grivas G., Athanasopoulou E., Kakouri A., Bailey J., Liakakou E., Stavroulas I., Kalkavouras P., Bougiatioti A., Kaskaoutis D.G., Ramonet M. (2020). Integrating in situ Measurements and City Scale Modelling to Assess the COVID–19 Lockdown Effects on Emissions and Air Quality in Athens, Greece. Atmosphere.

[65] Kerimray A., Baimatova N., Ibragimova O.P., Bukenov B., Kenessov B., Plotitsyn P., Karaca F. (2020). Assessing air quality changes in large cities during COVID-19 lockdowns: The impacts of traffic-free urban conditions in Almaty, Kazakhstan. Sci. Total Environ..

[66] Hudda N., Simon M., Patton A., Durant J. (2020). Reductions in traffic-related black carbon and ultrafine particle number concentrations in an urban neighborhood during the COVID-19 pandemic. Sci. Total Environ..

[67] Collivignarelli M.C., Abbà A., Bertanza G., Pedrazzani R., Ricciardi P., Carnevale Miino M. (2020). Lockdown for CoViD-2019 in Milan: What are the effects on air quality?. Sci. Total Environ..

[68] Chen Y., Zhang S., Peng C., Shi G., Tian M., Huang R.-J., Guo D., Wang H., Yao X., Yang F. (2020). Impact of the COVID-19 pandemic and control measures on air quality and aerosol light absorption in Southwestern China. Sci. Total Environ..

[69] Wang H., Miao Q., Shen L., Yang Q., Wu Y., Wei H., Yin Y., Zhao T., Zhu B., Lu W. (2021). Characterization of the aerosol chemical composition during the COVID-19 lockdown period in Suzhou in the Yangtze River Delta, China. J. Environ. Sci..

[70] Panda S., Mallik C., Nath J., Das T., Ramasamy B. (2020). A study on variation of atmospheric pollutants over Bhubaneswar during imposition of nationwide lockdown in India for the COVID-19 pandemic. Air Qual. Atmos. Heal..

[71] Petzold A., Ogren J.A., Fiebig M., Laj P., Li S.M., Baltensperger U., Holzer-Popp T., Kinne S., Pappalardo G., Sugimoto N. (2013). Recommendations for reporting black carbon measurements. Atmos. Chem. Phys..

[72] Marín J.C., Raga G.B., Arévalo J., Baumgardner D., Córdova A.M., Pozo D., Calvo A., Castro A., Fraile R., Sorribas M. (2017). Properties of particulate pollution in the port city of Valparaiso, Chile. Atmos. Environ..

[73] Virkkula A., Mäkelä T., Hillamo R., Yli-Tuomi T., Hirsikko A., Hämeri K., Koponen I.K. (2007). A simple procedure for correcting loading effects of aethalometer data. J. Air Waste Manag. Assoc..

[74] Drinovec L., Močnik G., Zotter P., Prévôt A.S.H., Ruckstuhl C., Coz E., Rupakheti M., Sciare J., Müller T., Wiedensohler A. (2015). The “dual-spot” Aethalometer: An improved measurement of aerosol black carbon with real-time loading compensation. Atmos. Meas. Tech..

[75] Hansen A.D.A. (2005). Magee Scientific Aethalometer Handbook, 2005.07.

[76] Liu Y., Yan C., Zheng M. (2018). Source apportionment of black carbon during winter in Beijing. Sci. Total Environ..

[77] Becerril-Valle M., Coz E., Prévôt A.S.H., Močnik G., Pandis S.N., Sánchez de la Campa A.M., Alastuey A., Díaz E., Pérez R.M., Artíñano B. (2017). Characterization of atmospheric black carbon and co-pollutants in urban and rural areas of Spain. Atmos. Environ..

[78] Omokungbe O.R., Fawole O.G., Owoade O.K., Popoola O.A.M., Jones R.L., Olise F.S., Ayoola M.A., Abiodun P.O., Toyeje A.B., Olufemi A.P. (2020). Analysis of the variability of airborne particulate matter with prevailing meteorological conditions across a semi-urban environment using a network of low-cost air quality sensors. Heliyon.

[79] Habeebullah T.M., Munir S., Awad A.H.A.A., Morsy E.A., Seroji A.R., Mohammed A.M.F. (2015). The Interaction between Air Quality and Meteorological Factors in an Arid Environment of Makkah, Saudi Arabia. Int. J. Environ. Sci. Dev..

[80] Nadzir M.S.M., Ooi M.C.G., Alhasa K.M., Bakar M.A.A., Mohtar A.A.A., Nor M.F.F.M., Latif M.T., Hamid H.H.A., Ali S.H.M., Ariff N.M. (2020). The impact of movement control order (MCO) during pandemic COVID-19 on local air quality in an urban area of Klang valley, Malaysia. Aerosol Air Qual. Res..

[81] Wang Y.Q., Zhang X.Y., Draxler R.R. (2009). TrajStat: GIS-based software that uses various trajectory statistical analysis methods to identify potential sources from long-term air pollution measurement data. Environ. Model. Softw..

[82] Anil I., Alagha O., Karaca F. (2017). Effects of transport patterns on chemical composition of sequential rain samples: Trajectory clustering and principal component analysis approach. Air Qual. Atmos. Heal..

[83] Anil I., Alagha O., Blaisi N.I., Mohamed I.A., Barghouthi M.H., Manzar M.S. (2019). Source Identification of Episodic Rain Pollutants by New Approach: Combining Satellite Observations and Backward Air Mass Trajectories. Aerosol Air Qual. Res..

[84] Ma P.L., Gattiker J.R., Liu X., Rasch P.J. (2013). A novel approach for determining source-receptor relationships in model simulations: A case study of black carbon transport in northern hemisphere winter. Environ. Res. Lett..

[85] Kumar R.R., Soni V.K., Jain M.K. (2020). Evaluation of spatial and temporal heterogeneity of black carbon aerosol mass concentration over India using three year measurements from IMD BC observation network. Sci. Total Environ..

[86] Zhuang B.L., Wang T.J., Liu J., Li S., Xie M., Yang X.Q., Fu C.B., Sun J.N., Yin C.Q., Liao J.B. (2014). Continuous measurement of black carbon aerosol in urban Nanjing of Yangtze River Delta, China. Atmos. Environ..

[87] Ji D., Li L., Pang B., Xue P., Wang L., Wu Y., Zhang H., Wang Y. (2017). Characterization of black carbon in an urban-rural fringe area of Beijing. Environ. Pollut..

[88] Helin A., Niemi J.V., Virkkula A., Pirjola L., Teinilä K., Backman J., Aurela M., Saarikoski S., Rönkkö T., Asmi E. (2018). Characteristics and source apportionment of black carbon in the Helsinki metropolitan area, Finland. Atmos. Environ..

[89] Liakakou E., Kaskaoutis D.G., Grivas G., Stavroulas I., Tsagkaraki M., Paraskevopoulou D., Bougiatioti A., Dumka U.C., Gerasopoulos E., Mihalopoulos N. (2020). Long-term brown carbon spectral characteristics in a Mediterranean city (Athens). Sci. Total Environ..

[90] Kharol S.K., Badarinath K.V.S., Sharma A.R., Mahalakshmi D.V., Singh D., Prasad V.K. (2012). Black carbon aerosol variations over Patiala city, Punjab, India—A study during agriculture crop residue burning period using ground measurements and satellite data. J. Atmos. Solar Terrestrial Phys..

[91] Mousavi A., Sowlat M.H., Lovett C., Rauber M., Szidat S., Boffi R., Borgini A., De Marco C., Ruprecht A.A., Sioutas C. (2019). Source apportionment of black carbon (BC) from fossil fuel and biomass burning in metropolitan Milan, Italy. Atmos. Environ..

[92] Xie M., Chen X., Holder A.L., Hays M.D., Lewandowski M., Offenberg J.H., Kleindienst T.E., Jaoui M., Hannigan M.P. (2019). Light absorption of organic carbon and its sources at a southeastern U.S. location in summer. Environ. Pollut..

[93] Begam G.R., Vachaspati C.V., Ahammed Y.N., Kumar K.R., Babu S.S., Reddy R.R. (2016). Measurement and analysis of black carbon aerosols over a tropical semi-arid station in Kadapa, India. Atmos. Res..

